# Factors associated with carotid intima-media thickness progression in patients with asymptomatic hyperuricemia: insights from the PRIZE study

**DOI:** 10.1038/s41598-023-37183-0

**Published:** 2023-07-05

**Authors:** Yuichi Saito, Atsushi Tanaka, Tomoko Ishizu, Hisako Yoshida, Yoshiaki Kubota, Mamoru Nanasato, Munehide Matsuhisa, Yusuke Ohya, Yoshio Kobayashi, Koichi Node, the PRIZE Study Investigators, Toyoaki Murohara, Teruo Inoue, Masataka Sata, Mitsuru Ohishi, Kotaro Yokote, Kazuomi Kario, Hirotaka Watada, Iichiro Shimomura, Munehide Matsuhisa, Yoshihiro Fukumoto, Koji Maemura, Yusuke Ohya, Yuichi Akasaki, Junya Ako, Hirohisa Amano, Kazutaka Aonuma, Yutaka Aoyama, Hirofumi Arai, Kuniya Asai, Machiko Asaka, Yoshifumi Awaji, Noriko Ban, Toshiaki Ban, Yasuko K. Bando, Hiroyuki Daida, Shunsuke Eguchi, Mami Enomoto, Yuichi Fujii, Akinori Fujikake, Masanori Fujimoto, Tomohiro Fujisaka, Shuichi Fujita, Satoki Fukae, Daiju Fukuda, Mieko Fukui, Yuhei Goriki, Shuichi Hamasaki, Tomoya Hara, Hiroshi Hasegawa, Kenichi Hashimoto, Mitsumasa Hata, Shiro Hata, Ryo Hayashida, Akihiro Higashi, Seiichiro Higuchi, Akihiro Honda, Satoshi Hoshide, Masaaki Hoshiga, Junko Hotchi, Sachiyo Igata, Yumi Ikehara, Teruo Inoue, Youhei Inoue, Hiroko Ishigami, Masaharu Ishihara, Hideki Ishii, Tetsuya Ishikawa, Takashi Ishimatsu, Yusuke Ishiyama, Takahide Ito, Ayumi Ito, Toshiaki Kadokami, Haruo Kamiya, Soichiro Kashihara, Yoshihiro Kawamura, Kazuo Kitagawa, Yoshio Kobayashi, Satoshi Kodera, Seiji Koga, Hisashi Koide, Yuji Koide, Hiroshi Koiwaya, Hiroki Kojima, Eri Komai, Takaaki Komatsu, Shingo Kono, Takashi Kono, Yoshiaki Kubota, Akio Kuroda, Takanori Kuroyanagi, Akifumi Kushiyama, Kenya Kusunose, Tatsuya Maruhashi, Kazuo Matsunaga, Tomomi Matsuura, Takafumi Mayama, Daigo Mine, Masatoshi Miyamura, Ryota Morimoto, Hideaki Morita, Hidekazu Nagano, Hidemitsu Nakagawa, Katsunori Nakamura, Ryo Nakamura, Ikuko Nakamura, Hitoshi Nakashima, Mamoru Nanasato, Isao Nishi, Shinichi Niwano, Shuichi Nomura, Nozomu Oda, Shio Oguchi, Mitsutoshi Oguri, Arihide Okahara, Masaaki Okutsu, Fumitake Ozaki, Michishige Ozeki, Tomoko Saisu, Yuichi Saito, Makoto Saitoh, Yosuke Saka, Yoshihiko Sakai, Kazushi Sakane, Ikki Sakuma, Shakya Sandeep, Hiroaki Sano, Hisakuni Sekino, Yuka Senoo, Kensaku Shibata, Yoshisato Shibata, Takahisa Shibata, Akina Shiga, Kazuki Shiina, Michio Shimabukuro, Yusaku Shimbo, Wataru Shimizu, Masahisa Shimpo, Takeshi Soeki, Koichi Sohmiya, Hiroyuki Suzuki, Susumu Suzuki, Makoto Suzuki, Nobuhiro Tahara, Tazu Tahara, Sadako Takahashi, Bonpei Takase, Kaoru Takegami, Tomoko Takiguchi, Tomonobu Takikawa, Ai Tamura, Tomoaki Tanaka, Akihito Tanaka, Hiroyuki Tanaka, Jun Tanigawa, Daisuke Tanimura, Yosuke Tatami, Takashi Terano, Fumio Terasaki, Tomoyuki Tobushi, Seiko Tokoi, Toshiyuki Tsubouchi, Daigaku Uchida, Tomohiro Ueda, Rie Ueno, Hiromi Ueno, Chikara Ueyama, Tetsuzo Wakatsuki, Tomohiko Watanabe, Masato Watarai, Isao Yaguchi, Ayumu Yajima, Jiko Yamada, Kyohei Yamamoto, Sachiko Yamauchi, Yohei Yamauchi, Naoto Yokota, Tomohiko Yoshida, Goro Yoshioka, Hiroyuki Daida, Junya Ako, Kazuo Kitagawa, Wataru Shimizu, Yoshio Kobayashi, Masaharu Ishihara, Tomoko Ishizu, Shinichiro Ueda, Atsushi Tanaka, Jun-ichi Oyama, Mikiko Kagiyama

**Affiliations:** 1 Department of Cardiovascular Medicine, Chiba University Graduate School of Medicine, Chiba, Japan; 2 Department of Cardiovascular Medicine, Saga University, Saga, Japan; 3 Department of Cardiology, Faculty of Medicine, University of Tsukuba, Tsukuba, Japan; 4 Department of Medical Statistics, Osaka Metropolitan University Graduate School of Medicine, Osaka, Japan; 5 Department of Cardiovascular Medicine, Nippon Medical School, Tokyo, Japan; 6 Department of Cardiology, Sakakibara Heart Institute, Fuchu, Japan; 7 Diabetes Therapeutics and Research Center, Institute of Advanced Medical Sciences, Tokushima University, Tokushima, Japan; 8 Department of Cardiovascular Medicine, Nephrology and Neurology, Graduate School of Medicine, University of the Ryukyus, Okinawa, Japan; 9 Nagoya University Graduate School of Medicine, Nagoya, Japan; 10 Dokkyo Medical University, Mibu, Japan; 11 Tokushima University Graduate School, Tokushima, Japan; 12 Kagoshima University, Kagoshima, Japan; 13 Chiba University Graduate School of Medicine, Chiba, Japan; 14 Jichi Medical University School of Medicine, Shimotsuke, Japan; 15 Juntendo University Graduate School of Medicine, Tokyo, Japan; 16 Osaka University, Graduate School of Medicine, Suita, Japan; 17 Kurume University School of Medicine, Kurume, Japan; 18 Nagasaki University Graduate School of Biomedical Sciences, Nagasaki, Japan; 19 University of the Ryukyus, Okinawa, Japan; 20 Kitasato University School of Medicine, Sagamihara, Japan; 21 Graduate School of Comprehensive Human Sciences, University of Tsukuba, Tsukuba, Japan; 22 Nagoya Daini Red Cross Hospital, Nagoya, Japan; 23 Kameda Medical Center, Komogawa, Japan; 24 Nippon Medical School, Tokyo, Japan; 25 Saga University, Saga, Japan; 26 Nagoya Ekisaikai Hospital, Nagoya, Japan; 27 Chiba Aoba Municipal Hospital, Chiba, Japan; 28 Isumi Medical Center, Isumi, Japan; 29 Japanese Red Cross Nagoya Daini Hospital, Nagoya, Japan; 30 Hiroshima General Hospital of West Japan Railway Company, Hiroshima, Japan; 31 Dokkyo Medical University Saitama Medical Center, Koshigaya, Japan; 32 Graduate School of Medicine, Chiba University, Chiba, Japan; 33 Osaka Medical College, Takatsuki, Japan; 34 Tokushima University Graduate School of Biomedical Sciences, Tokushima, Japan; 35 Kimitsu Chuo Hospital, Kisarazu, Japan; 36 Miyazaki Medical Association Hospital, Miyazaki, Japan; 37 Kagoshima City Hospital, Kagoshima, Japan; 38 National Defense Medical College, Tokorozawa, Japan; 39 Sekino Hospital, Tokyo, Japan; 40 Sasebo City General Hospital, Sasebo, Japan; 41 University of the Ryukyus, Nishihara, Japan; 42 Hyogo College of Medicine, Nishinomiya, Japan; 43 Fukuoka Saiseikai Futsukaichi Hospital, Chikushino, Japan; 44 Japanese Red Cross Nagoya Daiichi Hospital, Nagoya, Japan; 45 Kasugai Municipal Hospital, Kasugai, Japan; 46 Tokyo Women’s Medical University, Tokyo, Japan; 47 Asahi General Hospital, Asahi, Japan; 48 Kobe City Medical Center General Hospital, Kobe, Japan; 49 Institute of Advanced Medical Sciences, Tokushima University, Tokushima, Japan; 50 The Institute for Adult Diseases, Asahi Life Foundation, Tokyo, Japan; 51 Graduate School of Biomedical and Health Sciences, Hiroshima University, Hiroshima, Japan; 52 Imari Arita Kyoritsu Hospital, Matsuura, Japan; 53 Saga-Ken Medical Centre Koseikan, Saga, Japan; 54 Nozaki Tokushukai Hospital, Daito, Japan; 55 Ryukyu University Hospital, Nishihara, Japan; 56 National Hospital Organization Kagoshima Medical Center, Kagoshima, Japan; 57 National Hospital Organization Kasumigaura Medical Center, Tsuchiura, Japan; 58 Tokyo Medical University, Tokyo, Japan; 59 Chiba University Hospital, Chiba, Japan; 60 Nishio Municipal Hospital, Nishio, Japan; 61 Niko Clinic, Takeo, Japan; 62 Hotaruno Central Clinic, Kisarazu, Japan; 63 Gifu Prefectural Tajimi Hospital, Tajimi, Japan; 64 Anjo Kosei Hospital, Anjo, Japan; 65 Yokota Naika, Miyazaki, Japan; 66 Tsukuba, Japan; 67 Tsukuba University, Tsukuba, Japan; 68 Clinical Research Management Center, University of the Ryukyus, Okinawa, Japan; 69 Okinawa, Japan; 70 Tokyo, Japan

**Keywords:** Biomarkers, Endocrinology, Medical research, Risk factors

## Abstract

Hyperuricemia is reportedly associated with the progression of carotid intima-media thickness (IMT), a surrogate of cardiovascular risks and events. However, factors associated with carotid IMT progression in patients with asymptomatic hyperuricemia are largely unknown. In this post-hoc analysis of the multicenter, randomized PRIZE study, we analyzed data from a total of 326 patients who underwent carotid ultrasonography in a blind manner at baseline and 24 months to evaluate carotid IMT. Mean and maximum IMT at the common carotid artery (CCA) were measured at a central core laboratory. Factors related to the absolute change in mean and maximum IMT from baseline to 24 months were explored. Overall, the adjusted mean [0.0032 (− 0.0214 to 0.0278) mm] and maximum [0.0011 (− 0.0327 to 0.0351) mm] CCA-IMT increased numerically from baseline to 24 months. Multivariable analysis identified higher body mass index, history of atherosclerotic cardiovascular disease (ASCVD), and lower mean CCA-IMT at baseline as significant factors associated with the increase in mean CCA-IMT. In addition, older age and lower mean CCA-IMT at baseline were significant factors for an increased absolute change in the maximum CCA-IMT at 24 months. The present sub-analysis of the PRIZE study showed higher body mass index, history of ASCVD, and older age as significant factors associated with CCA-IMT progression in patients with asymptomatic hyperuricemia. These factors may be considered when identifying the possible risk of atherosclerotic progression in this specific patient population of hyperuricemia.

Trial registration: UMIN000012911 and UMIN000041322.

## Introduction

Uric acid has significant roles in gout and kidney stone formation. Beyond crystalline arthropathy and urolithiasis, an elevated serum uric acid (SUA) level may be associated with the development of cardiometabolic and cardiovascular diseases, such as hypertension, insulin resistance, chronic kidney disease, heart failure, and coronary artery disease^1^. Although a causal effect of hyperuricemia on atherosclerotic progression has long been a subject of investigation and debate, an elevated SUA level is at least considered an indicator of cardiovascular events^1^. In this context, elevated SUA levels are reportedly associated with carotid intima-media thickness (IMT) progression^2–4^, a surrogate marker of cardiovascular risk and events^5,6^, in healthy individuals and patients with several risk factors. In addition to conventional cardiovascular risk factors, including hypertension, hypercholesteremia, diabetes, and smoking, hyperuricemia has been shown to be an independent predictor of IMT progression^7–10^. However, few previous investigations have focused on hyperuricemic patients without gout, and predictors of IMT progression in this specific population are largely unknown. In the present study, we aimed to evaluate factors associated with carotid IMT progression in patients with asymptomatic hyperuricemia.

## Methods

### Study design and participants

This was a post-hoc analysis of the PRIZE (program of vascular evaluation under uric acid control by xanthine oxidase inhibitor, febuxostat: multicenter, randomized controlled) study, a prospective, open-label, blinded-endpoint clinical trial (University Hospital Medical Information Network Clinical Trial Registry; UMIN000012911 and UMIN000041322). The detailed study protocol and design are available in previous publications^11–14^. The study was conducted in accordance with the Declaration of Helsinki, and the study protocol was approved by the Ethics Committee Saga University Hospital (2013-10-02 and 2020-05-R01). Written informed consent for the PRIZE study was obtained from all participants.

**Figure 1:**
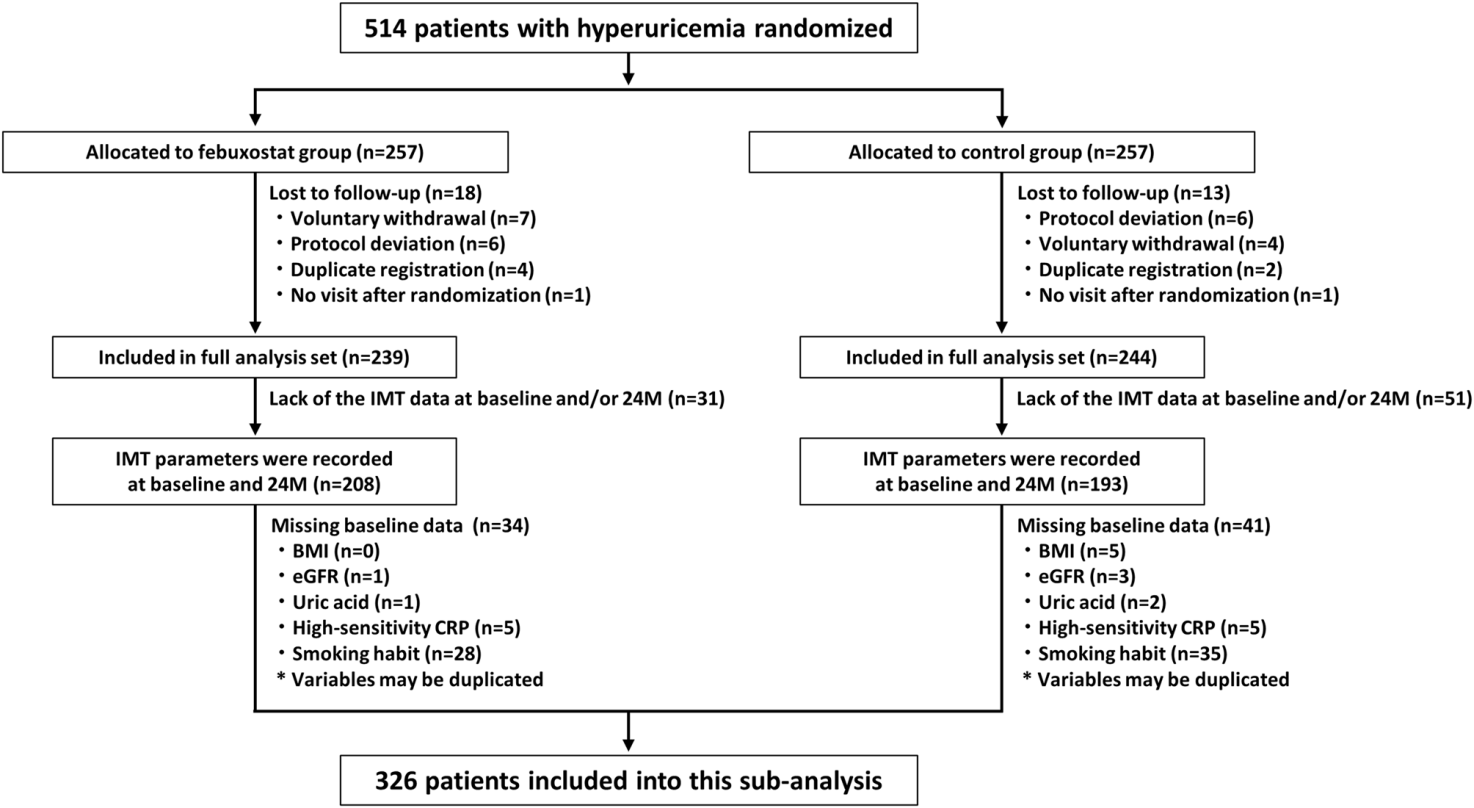
Study flow. *BMI* body mass index, *CRP* C-reactive protein, *eGFR* estimated glomerular filtration rate, *IMT* intima-media thickness.

Individuals were eligible for the PRIZE study when their SUA level was > 7.0 mg/dl and the maximum IMT of the common carotid artery (CCA) was ≥ 1.1 mm at screening. Patients who received SUA-lowering agents within the 8-week period prior to assessment of eligibility and had gouty tophus or symptoms of gouty arthritis within one year were excluded. Patents with hyperuricemia and not receiving SUA-lowering agents or showing significant carotid plaques were randomly allocated in a 1:1 ratio to receive febuxostat, a xanthine oxidase inhibitor, and appropriate lifestyle modification for hyperuricemia (febuxostat group) or lifestyle modification alone (control group). Randomization was stratified according to age (< 65 or ≥ 65 years), sex, diabetes, SUA level (< 8.0 or ≥ 8.0 mg/dl), and maximum CCA-IMT (< 1.3 or ≥ 1.3 mm). Participants allocated to receive febuxostat were treated with an initial dose of 10 mg daily, followed by an increase to 20 mg daily at one month and an increase to 40 mg daily at two months, if tolerated. Although a dose of 40 mg daily was targeted as the maintenance dose, it could be increased up to 60 mg daily at three months or later. Major exclusion criteria for the present sub-analysis were lack of carotid IMT measurement at baseline and/or 24 months and missing baseline data (Fig. 1). Thus, a total of 326 patients were included in this sub-analysis.

### Carotid IMT measurement

According to the standard protocol, the carotid IMT images were recorded by a dedicated sonographer using high-resolution carotid ultrasonography with a > 7.5-MHz liner transducer at each study site and were read by an experienced analyzer at a central core laboratory in a blinded manner. IMT measurement was performed in accordance with the Japan Society of Ultrasonics in Medicine and the Japan Academy of Neurosonology using an automated IMT measurement software program (Vascular Research Tools 5, Medical Imaging Applications LLC, Coralville, USA)^15^. Longitudinal B-mode images of each CCA were obtained, and mean CCA-IMT was determined within a 10-mm region proximal to the origin of the bulb to obtain the averaged thickness of carotid plaque. Maximum CCA-IMT was also evaluated within the region. The mean and maximum IMT values on the left and right sides were averaged at baseline and at 24 months. The absolute change from baseline to 24 months was calculated.

### Definitions

In the present analysis, hypertension was defined as having a previous diagnosis of hypertension (office systolic blood pressure ≥ 140 mm Hg and/or diastolic blood pressure ≥ 90 mm Hg) or previous antihypertensive medications, according to the guidelines^16^. Diabetes was defined as a previous diagnosis of diabetes (glycated hemoglobin ≥ 6.5%, fasting plasma glucose ≥ 126 mg/dl, or 2-h plasma glucose after 75-g oral glucose tolerance test ≥ 200 mg/dl, or a random plasma glucose ≥ 200 mg/dl) or previous glucose-lowering medications, based on the guidelines^17^. Dyslipidemia was defined as a previous diagnosis of dyslipidemia or previous lipid-lowering medications. Atherosclerotic cardiovascular disease (ASCVD) included coronary artery disease (a composite of a previous diagnosis of angina pectoris and myocardial infarction or a history of percutaneous coronary intervention and coronary artery bypass grafting) and cerebrovascular disease (a previous diagnosis of ischemic stroke and transient ischemic attack). Patients were divided into two groups according to age (≥ 75 vs. < 75 years), body mass index (BMI) (≥ 25 vs. < 25), and the presence or absence of ASCVD.

### Study endpoint and statistical analysis

The primary endpoint of this sub-analysis was the absolute change in mean CCA-IMT from baseline to 24 months. The absolute change in maximum CCA-IMT from baseline to 24 months was also evaluated. Factors associated with IMT progression in patients with hyperuricemia were explored.

Statistical analysis was performed using R statistical software version 4.0.2 (R Foundation for Statistical Computing, Vienna, Austria). All continuous data are expressed as median [interquartile range], and categorical data are shown as frequency (%). Multivariable analysis was performed using a multiple linear regression model for identifying factors associated with the absolute change in mean and maximum CCA-IMT from baseline to 24 months. Non-standardized and standardized regression coefficients were evaluated, and an adjusted change in CCA-IMT from baseline to 24 months with a 95% confidence interval was determined. Twelve factors reportedly associated with IMT progression, including age, sex, BMI, hypertension, diabetes, dyslipidemia, smoking, a history of ASCVD, renal function (assessed with estimated glomerular filtration rate), SUA level, high-sensitivity C-reactive protein level, and baseline mean CCA-IMT were included into the multivariable model in addition to the study allocation^3,4,18–27^. Because the C-reactive protein level was not normally distributed, it was log-transformed in the multivariable analysis. A *p*-value of < 0.05 was considered statistically significant.

### Ethics approval and consent to participate

The study was conducted in accordance with the Declaration of Helsinki, and the study protocol was approved by the local institutional review boards and independent ethic committees at all sites. Written informed consent for the PRIZE study was obtained from all participants.

## Results

From May 2014 to June 2016, a total of 514 patients were enrolled in the PRIZE study and were randomized to either the febuxostat group (n = 257) or the control group (n = 257), among whom 326 (63.4%) were included in the present analysis. Baseline characteristics are shown in Table 1. Median age was 70 [63, 76] years, men accounted for more than 80% of the analysis population, and cardiovascular risk factors were prevalent. More than one-third of the population had a history of ASCVD, and patients were well treated with medications, including antiplatelet and lipid-lowering agents (Table 1). During the study period, 3 of 326 (0.9%) patients developed gout, all of whom were in the control group.

**Table 1:** Baseline characteristics.

Variable	All patients (n = 326)
Age, years	70 [63, 76]
Men	271 (83.1)
Body mass index, kg/m^2^	24.7 [22.6, 27.1]
Hypertension	292 (89.6)
Diabetes	126 (38.7)
Dyslipidemia	194 (59.5)
Current smoking	41 (12.6)
ASCVD	114 (35.0)
Coronary artery disease	97 (29.8)
Cerebrovascular disease	22 (6.7)
eGFR, ml/min/1.73 m^2^	55.0 [46.2, 66.7]
Serum uric acid, mg/dl	7.6 [7.1, 8.2]
hs-CRP, ng/ml	670.0 [340.8, 1630.0]
Medications at baseline	
Antiplatelet agent	140 (42.9)
ACE-I or ARB	220 (67.5)
Calcium channel blocker	182 (55.8)
β-blocker	127 (39.0)
Diuretic	101 (31.0)
Statin	162 (49.7)
Ezetimibe	9 (2.8)
Oral hypoglycemic agent	102 (31.3)
Insulin	17 (5.2)

Values are expressed as median [interquartile range] or number (%).

*ACE-I* angiotensin converting enzyme inhibitor, *ARB* angiotensin II receptor blocker, *ASCVD* atherosclerotic cardiovascular disease, *hs-CRP* high-sensitivity C-reactive protein, *eGFR* estimated glomerular filtration rate.

Overall, the mean [from 0.81 (0.73, 0.93) mm to 0.81 (0.72, 0.93) mm] and maximum [from 1.01 (0.91, 1.16) mm to 1.01 (0.89, 1.17) mm] CCA-IMT did not changed significantly from baseline to 24 months. Overall, the adjusted mean [0.0032 (− 0.0214 to 0.0278) mm] and maximum [0.0011 (− 0.0327 to 0.0351) mm] CCA-IMT increased numerically from baseline to 24 months.

**Figure 2:**
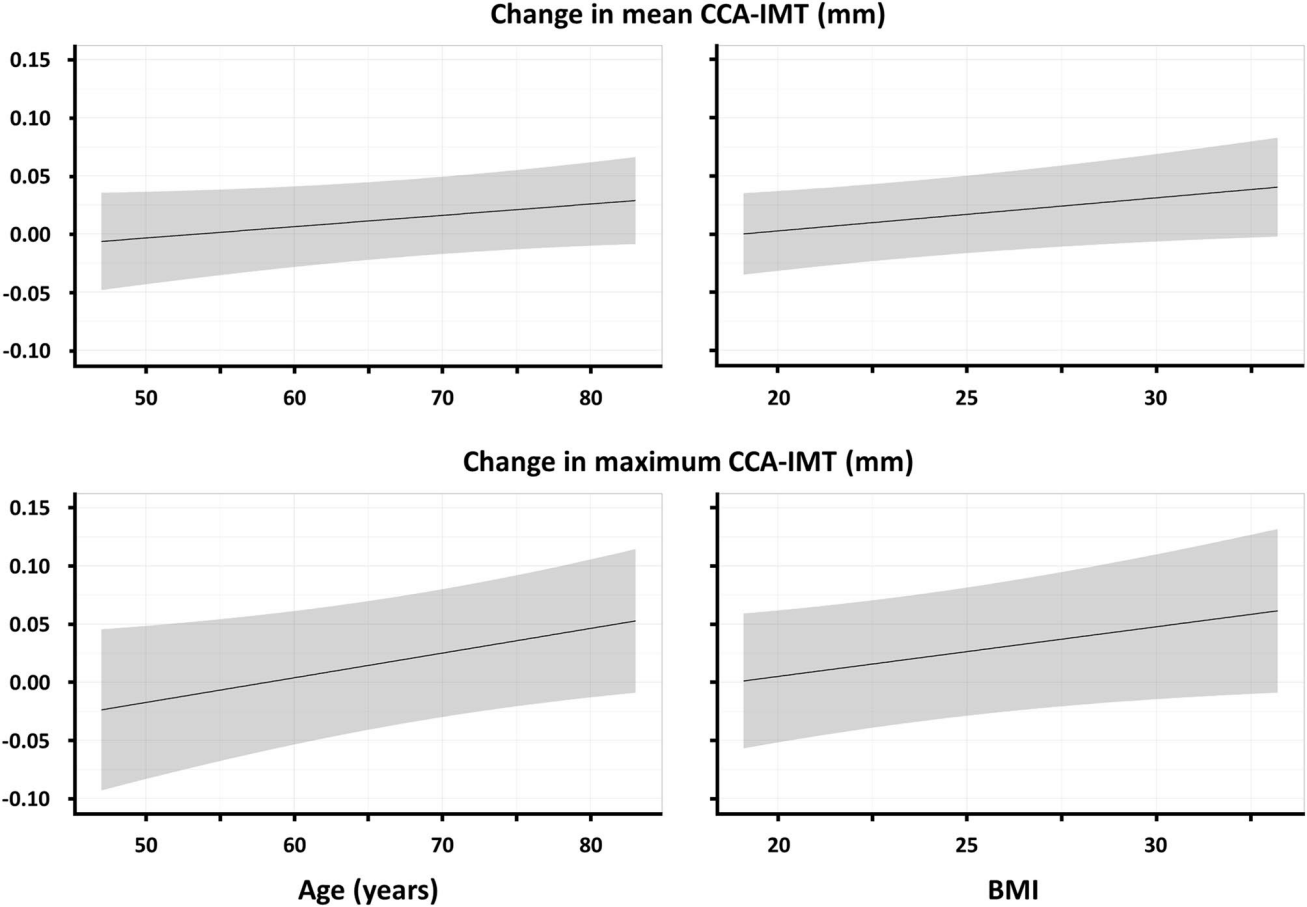
Relation of age and BMI to change in mean and maximum CCA-IMT. *BMI* body mass index, *CCA* common carotid artery, *IMT* intima-media thickness.

Multivariable analysis identified higher BMI, a history of ASCVD, and a lower mean CCA-IMT at baseline as significant factors associated with an increased absolute change in mean CCA-IMT from baseline to 24 months (Table 2 and Table S1). In addition, older age and a lower mean CCA-IMT at baseline were significant risk factors for an increased absolute change in maximum CCA-IMT (Table 3 and Table S2). In the multivariable analysis for the increase in maximum CCA-IMT, higher BMI and a history of ASCVD were not significantly associated with IMT progression (Table 3 and Table S2). Figure 2 displays the correlations of mean and maximum CCA-IMT with age and BMI. The adjusted absolute difference of change in mean and maximum CCA-IMT in age, BMI, and ASCVD categories are shown in Table S3 and S4. Patients aged ≥ 75 years with BMI ≥ 25 and ASCVD (n = 23) had an increase in mean CCA-IMT at 24 months [0.053 (0.009 to 0.097) mm], while mean CCA-IMT was not significantly changed during the follow-up period in those aged < 75 years with BMI < 25 and no ASCVD (n = 86) [0.003 (− 0.032 to 0.038) mm], with a significant between-group difference [0.050 (0.013 to 0.087) mm, p = 0.008].

**Table 2:** Multivariable analysis for the change in mean CCA-IMT.

Variable	Univariable		Multivariable	
	Regression coefficient	*P* value	Regression coefficient	*P* value
Age, per 10 years	− 0.001	0.82	0.010	0.11
Men	0.010	0.45	0.011	0.42
Body mass index, kg/m^2^	0.002	0.12	0.003	0.04
Hypertension	− 0.008	0.61	− 0.011	0.47
Diabetes	− 0.003	0.78	0.001	0.93
Dyslipidemia	− 0.008	0.40	− 0.008	0.45
Current smoking	− 0.024	0.11	− 0.022	0.14
ASCVD	0.008	0.46	0.023	0.04
eGFR, per 10 ml/min/1.73 m^2^	0.005	0.15	0.005	0.16
Serum uric acid, mg/dl	− 0.006	0.25	− 0.005	0.35
Log-hs-CRP, ng/ml	− 0.006	0.42	− 0.006	0.14
Baseline mean CCA-IMT, mm	− 0.010	< 0.001	− 0.012	< 0.001
Allocation to febuxostat group	− 0.005	0.61	− 0.003	0.79

*ASCVD* atherosclerotic cardiovascular disease, *CCA* common carotid artery, *eGFR* estimated glomerular filtration rate, *hs-CRP* high-sensitivity C-reactive protein, *IMT* intima-media thickness.

**Table 3:** Multivariable analysis for the change in maximum CCA-IMT.

Variable	Univariable		Multivariable	
	Regression coefficient	*P* value	Regression coefficient	*P* value
Age, per 10 years	0.004	0.62	0.021	0.03
Men	0.009	0.69	0.013	0.56
Body mass index, kg/m^2^	0.004	0.11	0.004	0.06
Hypertension	− 0.033	0.21	− 0.040	0.13
Diabetes	0.016	0.34	0.020	0.25
Dyslipidemia	− 0.005	0.78	− 0.005	0.77
Current smoking	− 0.029	0.23	− 0.020	0.42
ASCVD	0.017	0.31	0.035	0.06
eGFR, per 10 ml/min/1.73 m^2^	0.005	0.38	0.010	0.15
Serum uric acid, mg/dl	− 0.009	0.27	0.009	0.15
Log-hs-CRP, ng/ml	− 0.001	0.88	− 0.006	0.37
Baseline mean CCA-IMT, mm	− 0.012	< 0.001	− 0.015	< 0.001
Allocation to febuxostat group	− 0.017	0.31	− 0.014	0.39

*ASCVD* atherosclerotic cardiovascular disease, *CCA* common carotid artery, *eGFR* estimated glomerular filtration rate, *hs-CRP* high-sensitivity C-reactive protein, *IMT* intima-media thickness.

## Discussion

In the multicenter, prospective, randomized, controlled PRIZE study, mean and maximum CCA-IMT were analyzed in patients with asymptomatic hyperuricemia in a blinded manner at a central core laboratory at baseline and 24 months. In the present post-hoc analysis, higher BMI, a history of ASCVD, and older age were identified as factors related to IMT progression, confirming the importance of conventional risk markers in this specific population. To our knowledge, this is the first study to investigate factors associated with the development of carotid atherosclerosis in asymptomatic hyperuricemic patients.

Carotid IMT, the thickness of the intimal and medial layer of the carotid artery wall, can be non-invasively measured by ultrasound imaging and has been used as a surrogate marker of early-stage atherosclerosis in daily practice and clinical studies^5^. In general, among numerous previous studies, mean CCA-IMT progression reportedly ranges from 0 to 30 μm per year^25,27^. The European guidelines do not recommend systematic use of carotid IMT to improve risk assessment because of the lack of methodological standardization and the uncertainty of the added value of IMT in predicting future cardiovascular events^28^. However, a recent meta-analysis of 119 randomized, controlled trials including 100,667 patients showed that carotid IMT progression was significantly associated with an increased risk of cardiovascular disease, and effects of interventions on carotid IMT regression or delayed progression improved outcomes^5^, supporting the usefulness of IMT on carotid ultrasonography as a surrogate marker in clinical trials, as in the PRIZE study. Previous studies have indicated that conventional cardiovascular risk factors, such as hypertension, diabetes, dyslipidemia, current smoking, and obesity, are related to carotid IMT progression in various populations^5,27,29^, and elevated SUA levels may be associated with the development of carotid atherosclerosis^3,4,7–10^. However, no studies have focused on patients with an elevated SUA level in investigating IMT progression.

A meta-analysis of 15 cross-sectional studies including 11,382 participants showed that SUA levels correlated positively with carotid IMT^4^, but the present analysis and the original PRIZE study did not demonstrate a significant relationship between baseline SUA level and SUA-lowering therapy with febuxostat and carotid IMT progression. In a previous prospective longitudinal study that included patients with multiple cardiovascular risk factors in which core laboratory analysis was performed on carotid ultrasonography, faster IMT progression was observed in the group with elevated SUA levels (> 4.8 mg/dl for women and > 5.7 mg/dl for men) compared to their counterparts^3^. Given that the present analysis included only patients with elevated SUA levels (i.e. > 7.0 mg/dl), the results may be reasonable. In a previous single-center, cross-sectional study, the presence of hyperuricemia was significantly associated with the increase in carotid IMT in patients with essential hypertension, particularly in women^30^. A meta-analysis showed that there was a significant correlation between carotid-femoral pulse wave velocity and SUA levels in hypertensive women but not in men^31^. Because men accounted for more than 80%, and approximately 90% of patients had hypertension in the present study, the association between elevated levels of SUA and IMT progression may have been unclear. In addition, in a healthy population, the relation of SUA to cardiovascular organ damage including carotid plaque formation remains to be established^32^.

Nevertheless, because patients with hyperuricemia are at a high risk of cardiovascular events^1^, the identification of factors associated with the development of atherosclerosis is essential. In the present analysis, higher BMI, a history of ASCVD, and older age were identified as factors associated with IMT progression, while cardiovascular risk factors such as hypertension, diabetes, and dyslipidemia were not. It is important that the present study indicated higher BMI as a predictor of IMT progression, because obesity and hyperuricemia are closely associated based on both genetic and environmental factors^33^. Given that obesity is a modifiable risk factor and weight loss ameliorates cardiovascular risk, lifestyle intervention should be considered in patients with hyperuricemia to delay atherosclerosis^34,35^. In contrast to BMI, a history of ASCVD and older age are not modifiable. However, the identification of patients at high risk for IMT progression and future cardiovascular events may lead to intensive therapy, presumably resulting in improved clinical outcomes^36,37^. Interestingly, in addition to those three predictors, lower IMT at baseline was counterintuitively associated with IMT progression in the present study^38^, which may be explained by the fact that our study cohort had a relatively thin carotid plaques. Further studies are needed to clarify the impact of higher BMI, a history of ASCVD, and older age on carotid IMT progression and cardiovascular events in this specific population of patients with hyperuricemia.

The present analysis has several limitations. The PRIZE study was a prospective, randomized, controlled study, but the present analysis was conducted in a post-hoc fashion. Because most of participants in this study were Japanese, caution is warranted to interpret the results given the possible racial difference in the progression of carotid IMT^39^. A certain proportion of patients did not have IMT data at 24 months, resulting in the relatively small sample size. Nonetheless, standardized analysis of carotid ultrasonography at a central core laboratory was a study strength. Despite the entry criterion of CCA-IMT ≥ 1.1 mm, which may have affected the results and was measured at each participating hospital, the median maximum CCA-IMT at baseline evaluated by a core laboratory was < 1.1 mm, reinforcing the importance of organized and standardized IMT measurement. Since all patients included in the present study were asymptomatically hyperuricemic and none of them had a normal level of SUA at baseline, whether our results can be applied to populations with a normal SUA level is uncertain.

## Conclusions

The present sub-analysis of the PRIZE study identified higher BMI, a history of ASCVD, and older age as factors associated with carotid IMT progression in patients with asymptomatic hyperuricemia. These factors may be considered when identifying the possible risk of atherosclerotic progression in this specific patient population of hyperuricemia.

## Supplementary Information

Supplementary Table S1.

Supplementary Table S2.

Supplementary Table S3.

Supplementary Table S4.

## Data Availability

The datasets used and/or analysed during the current study available from the corresponding author on reasonable request.

## Abbreviations

ASCVD: Atherosclerotic cardiovascular disease
BMI: Body mass index
CCA: Common carotid artery
IMT: Intima-media thickness
SUA: Serum uric acid
