# Supplementary material for: Factors associated with carotid intima-media thickness progression in patients with asymptomatic hyperuricemia: insights from the PRIZE study
Source: Sci Rep. 2023 Jul 5;13:10927. doi: 10.1038/s41598-023-37183-0 (PMC10322958; doi:10.1038/s41598-023-37183-0)
Supplement: Supplementary file 2 — Supplementary Table S2. [file 41598_2023_37183_MOESM2_ESM.docx]

**Table S2. Multivariable Analysis for Change in the Maximum CCA-IMT**

| Variable | Multivariable | |
| --- | --- | --- |
|  | Standardized regression coefficient | *P* value |
| Age, per 1 SD (9.9 years) | 0.144 | 0.03 |
| Men | 0.034 | 0.56 |
| Body mass index, per 1 SD (3.7 kg/m^2^) | 0.109 | 0.06 |
| Hypertension | −0.083 | 0.13 |
| Diabetes | 0.068 | 0.25 |
| Dyslipidemia | −0.018 | 0.77 |
| Current smoking | −0.045 | 0.42 |
| ASCVD | 0.116 | 0.06 |
| eGFR, per 1 SD (15.6 ml/min/1.73 m^2^) | 0.099 | 0.15 |
| Serum uric acid, per 1 SD (0.9 mg/dl) | −0.055 | 0.35 |
| Log-hs-CRP, per 1 SD (1.1 ng/ml) | −0.050 | 0.37 |
| Baseline mean CCA-IMT, per 1 SD (0.2 mm) | −0.236 | <0.001 |
| Allocation to febuxostat group | −0.047 | 0.39 |

ASCVD, atherosclerotic cardiovascular disease; CCA, common carotid artery; eGFR, estimated glomerular filtration rate; hs-CRP, high-sensitivity C-reactive protein; IMT, intima-media thickness; SD, standard deviation.
