# Supplementary material for: Factors associated with carotid intima-media thickness progression in patients with asymptomatic hyperuricemia: insights from the PRIZE study
Source: Sci Rep. 2023 Jul 5;13:10927. doi: 10.1038/s41598-023-37183-0 (PMC10322958; doi:10.1038/s41598-023-37183-0)
Supplement: Supplementary file 3 — Supplementary Table S3. [file 41598_2023_37183_MOESM3_ESM.docx]

**Table S3. Adjusted Absolute Difference of Change in Mean CCA-IMT**

| Variable | Absolute difference (mm) (95% CI) | *P* value |
| --- | --- | --- |
| Age ≥75 vs. <75 years | 0.015 (−0.008 to 0.038) | 0.19 |
| Body mass index ≥25 vs. <25 | 0.012 (−0.008 to 0.032) | 0.23 |
| ASCVD vs. no ASCVD | 0.022 (0.000 to 0.045) | 0.06 |

ASCVD, atherosclerotic cardiovascular disease; CCA, common carotid artery; CI, confidence interval; IMT, intima-media thickness.
